# Effect of Traditional Chinese Medicine on COVID-19 Treatment: A Meta-Analysis of Randomized Clinical Trials

**DOI:** 10.3390/ph18030357

**Published:** 2025-03-02

**Authors:** Marharyta Sobczak, Rafał Pawliczak

**Affiliations:** Department of Immunopathology, Division of Biomedical Science, Faculty of Medicine, Medical University of Lodz, 90-752 Lodz, Poland; marharyta.sobczak@umed.lodz.pl

**Keywords:** traditional Chinese medicine, herbal medicine, COVID-19, coronavirus infection, meta-analysis

## Abstract

**Background/Objectives**: Traditional Chinese medicine (TCM) has a long history and is known for its anti-inflammatory, antiviral, and immunoregulatory qualities. It has been extensively studied during the COVID-19 pandemic. Therefore, to evaluate the relationship between TCM and the treatment of COVID-19, we conducted a meta-analysis. **Methods**: Our meta-analysis included 22 randomized clinical trials, which investigated the analyzed endpoints: time to recovery from fever, severity of dyspnea or breathlessness according on different scales, time to recovery for coughing, including dry and wet coughing, time to recovery for fatigue, changes in respiratory rate, length of hospitalization, hospital discharging rate, number of intensive care unit (ICU) admissions, number of cases requiring any supplemental oxygenation, number of deaths among COVID-19 patients, conversion rate of SARS-CoV-2 tests on a particular day, and time to viral assay conversion. The relative risk (RR) with 95% confidence intervals (CIs) and the mean difference or standardized mean difference with 95% CIs were calculated to compare the effect. A random effects model was used to calculate effect sizes. **Results**: We indicated a positive effect of TCM on different COVID-19 symptoms. TCM influences hospitalization duration, ICU admission, mortality, and time to viral assay conversion among COVID-19 patients. Moreover, TCM positively affects SARS-CoV-2 test conversion rates on particular days (RR = 1.21; 95% CI [1.10; 1.32]; *p* < 0.0001; I^2^ = 84%). **Conclusions**: TCM may potentially support the standard treatment of COVID-19. Nevertheless, the necessity for further randomized trials with a greater number of participants and in a wider range of countries remains apparent.

## 1. Introduction

One of the earliest civilizations in the world was the Chinese. Traditional Chinese medicine (TCM) has undergone a long process of development through the accumulation of practical experience, and its theory is constantly being refined. Despite the fact that it is practiced in many countries and has been suggested to have great potential for improving people’s health and well-being, there are still questions about its effectiveness. It was believed that the TCM theory was difficult to prove using modern scientific methods [1]. TCM traditionally consists of a number of different herbs or compounds with known or unknown active ingredients, which can be used for a variety of medical indications and are tailored to a person’s symptoms. Over the course of China’s long history, TCM has proven itself to treat infectious diseases and has been used to treat pandemic and endemic diseases. For example, during the 2002–2003 SARS-CoV outbreak in China, the use of TCM resulted in shorter hospitalization, reduced side effects of steroid treatment, and relief of breathlessness and malaise. It has been demonstrated that TCM exerts antiviral properties against a range of viral strains, including influenza virus, herpes simplex virus, human immunodeficiency virus, and hepatitis B and C viruses, as well as SARS-CoV and MERS-CoV [2].

According to TCM, there are two ideological concepts that serve as fundamental tenets. The first is the notion of homeostasis, which emphasizes the integrity of the human body and underscores the symbiotic relationship between the human organism and its social and natural environment. The second is the concept of dynamic balance, which prioritizes movement within integrity. In addition, TCM has its own unique understanding of the human body as well as disorders of the human body [3]. Herbal Chinese medicine dates back to the discovery of “Ma Huang” (herba ephedrae) around 3000 BC, which among other things was used to treat respiratory disorders [4]. Among the 12,807 types of Chinese medical materials, there are 11,146 types of plant medicine, 1581 types of animal medicine, 5000 types of Chinese patent medicine, 80 types of mineral medicine, and more than one million prescriptions. In addition to herbal medicine, there is Acu-moxa therapy (“zhen jiu”), which includes several techniques aimed at stimulating acupuncture points located on the body along circulatory pathways or conduits. The most popular of these techniques are acupuncture and moxibustion [5].

TCM has been found to have anti-inflammatory, antiviral, and immunoregulatory properties [6]. Active ingredients in TCM formulations that are effective in treating COVID-19 were identified using network pharmacology analysis. For example, it has the ability to target Mpro (SARS-CoV-2 3CL hydrolase), the main protease for SARS-CoV-2, or ACE2 (angiotensin-converting enzyme 2), the receptor for SARS-CoV-2, which may inhibit the development of COVID-19 [2,6]. In the context of the efforts to combat the novel coronavirus (SARS-CoV-2) in China, TCM has been formally incorporated into the third to eighth editions of the diagnostic and treatment guidelines promulgated by the National Health Commission of China [2]. Therefore, we decided to conduct a meta-analysis of randomized controlled trials (RCTs) to evaluate the relationship between TCM and the treatment of COVID-19.

## 2. Results

### 2.1. Search Results

**Figure 1:**
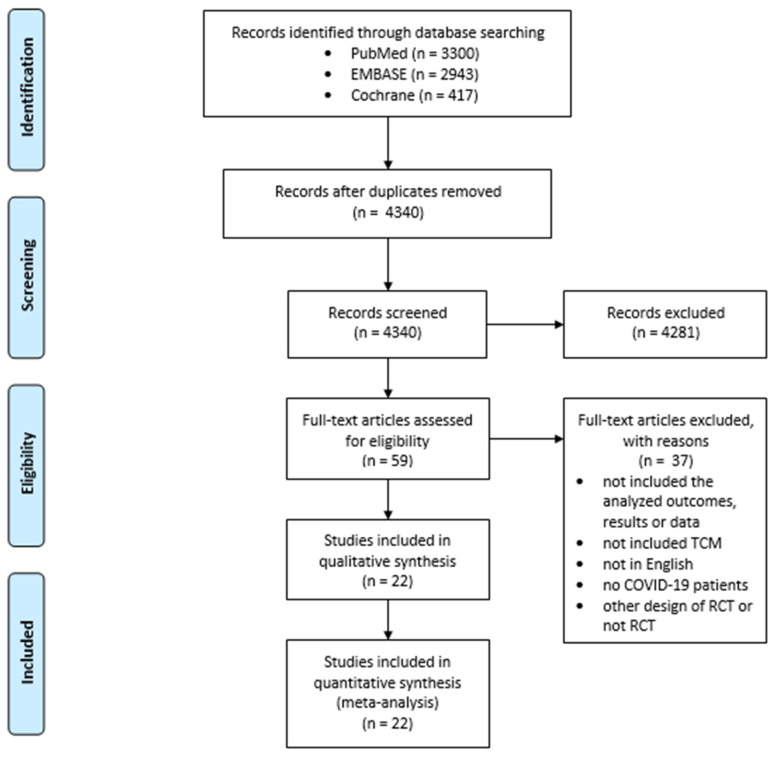
Selection of studies for meta-analysis.

The literature search yielded a total of 4340 articles, subsequent to the removal of duplicates (Figure 1). During the first screening process, 4281 articles were excluded on the basis of their categorization as meta-analyses, systematic reviews, literature reviews, editorial letters, in vitro studies, studies on animals, case reports, and observational studies. Furthermore, articles written in languages other than English were excluded. Following the full-text screening, 22 articles were deemed eligible for meta-analysis.

All included studies are randomized controlled trials with control groups, investigating the effect of TCM in COVID-19 treatment, which were carried out in China [7,8,9,10,11,12,13,14,15,16,17,18,19,20,21,22,23,24,25,26,27], except one study from Iran [28]. Three studies had placebos in the control group [21,22,26], and one study had a compound pholcodine oral solution in the control group [20]. Most of the studies had standard treatment in the control group and standard treatment with TCM in the experimental group [7,8,9,10,12,13,14,15,16,17,18,19,23,24,25,27,28]. In the study by Yu et al. [11], we considered the group that received Paxlovid (300 mg of Nirmatrelvir plus 100 mg of Ritonavir) as the control group, and the other 2 groups as experimental. Two studies included pediatric patients [20,24]. The characteristics of the included studies are shown in Table 1, while Table S1 shows the detailed description and formulas of TCM in the included studies.

### 2.2. Quality Evaluation

The risk of bias was determined for 22 included studies. Ten of the studies have a high risk of bias, while twelve indicate low risk. Figure S1 shows the summary of the risk of bias assessment.

### 2.3. Effect of TCM on Systemic and Respiratory Symptoms of COVID-19 and Changes in Respiratory Rate

**Figure 2:**
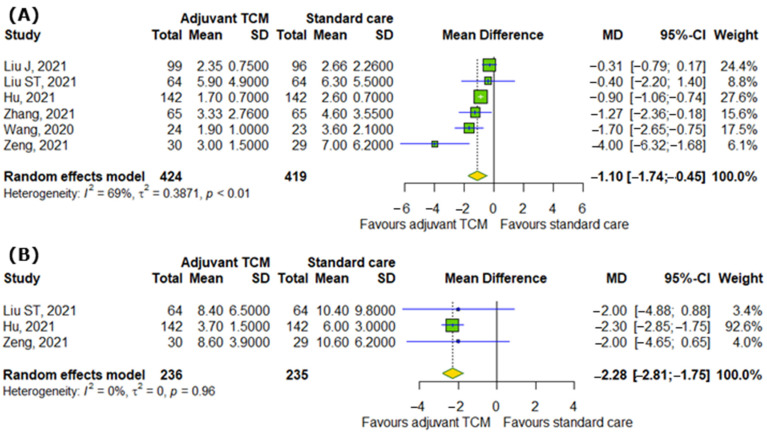
In comparison to standard COVID-19 treatment, the addition of traditional Chinese medicine to standard COVID-19 treatment affects systemic symptoms of COVID-19 by reducing (**A**) time to recovery from fever and (**B**) time to recovery from fatigue [8,9,13,16,17,25].

We analyzed the effect of TCM on symptoms of COVID-19 and changes in respiratory rate. Our analyzed showed the significant differences between interventional and control groups in systemic symptoms, such as time to recovery of fever (MD = −1.1; 95% CI [−1.74; −0.45]; *p* = 0.0009; I^2^ = 69%) and time to recovery for fatigue (MD = −2.28; 95% CI [−2.81; −1.75]; *p* < 0.0001; I^2^ = 0%), as represented in Figure 2A,B.

**Figure 3:**
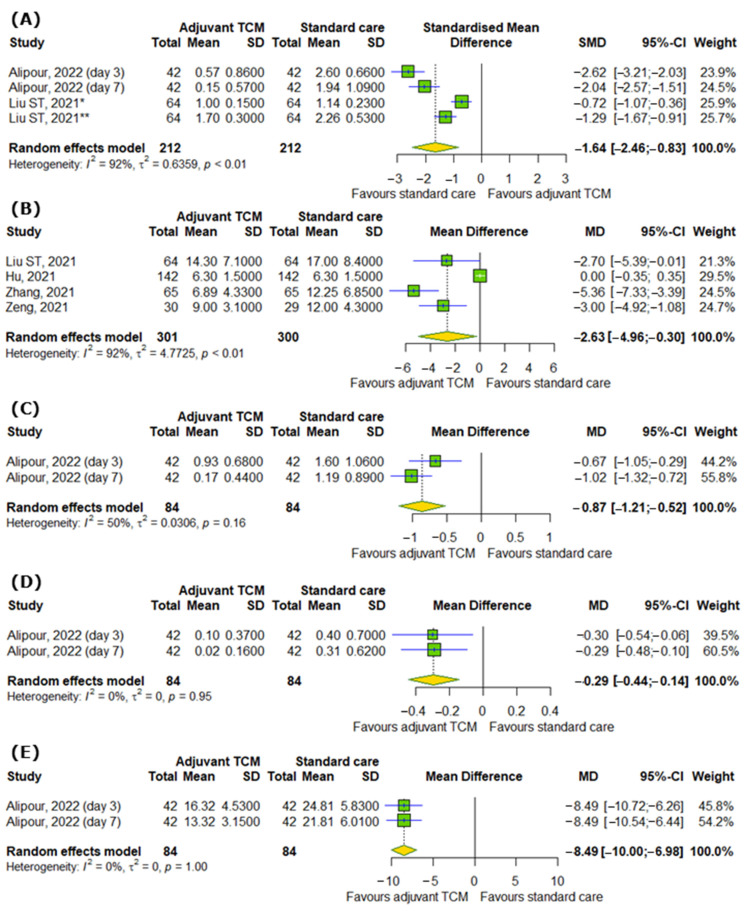
In comparison to standard COVID-19 treatment, the addition of traditional Chinese medicine to standard COVID-19 treatment affects respiratory symptoms of COVID-19 by reducing (**A**) the severity of dyspnea or breathlessness according to different scales, (**B**) time to recovery for coughing, including (**C**) dry and (**D**) wet coughing, and (**E**) respiratory rate [28]. * Medical Research Council (mMRC) dyspnea scale; ** modified Borg dyspnea scale.

In the case of respiratory symptoms, there were also significant differences between interventional and control groups, for example, the severity of dyspnea or breathlessness according to different scales (SMD = −1.64; 95% CI [−2.46; −0.83]; *p* < 0.0001; I^2^ = 92%); time to recovery for coughing (MD = −2.63; 95% CI [−4.96; −0.3]; *p* = 0.0272; I^2^ = 92%), including dry (MD = −0.87; 95% CI [−1.21; −0.52]; *p* < 0.0001; I^2^ = 50%) and wet coughing (MD = −0.29; 95% CI [−0.44; −0.14]; *p* = 0.0001; I^2^ = 0%); and changes in respiratory rate (MD = −8.49; 95% CI [−10.0; −6.98]; *p* < 0.0001; I^2^ = 0%), as represented in Figure 3A–E.

### 2.4. Effect of TCM on COVID-19 Hospitalization

**Figure 4:**
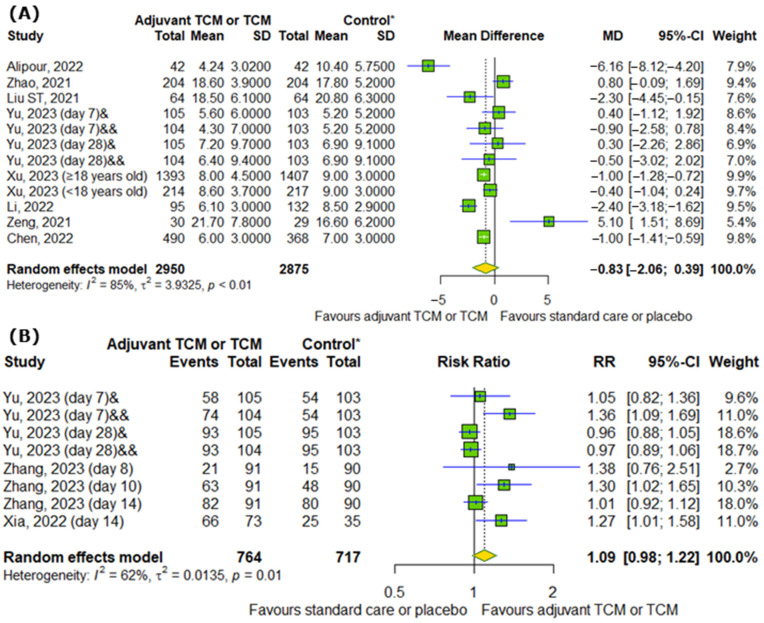
In comparison to standard COVID-19 treatment or placebo, the addition of traditional Chinese medicine to standard COVID-19 treatment or only TCM does not affect (**A**) COVID-19 hospitalization duration and (**B**) the discharge rates of COVID-19 patients on particular days [7,9,11,15,18,22,23,25,26,28]. * standard care or placebo; & Huashi Baidu; && Huashi Baidu and Paxlovid.

We analyzed the effect of TCM on COVID-19 hospitalization. Figure 4A showed that there are no differences between experimental and control groups in hospitalization duration (MD = −0.83; 95% CI [−2.06; 0.39]; *p* = 0.1827; I^2^ = 85%). Moreover, TCM did not significantly influence the discharge rates of hospitalized patients on particular days (RR = 1.09; 95% CI [0.98; 1.22]; *p* = 0.0962; I^2^ = 62%), as shown in Figure 4B.

### 2.5. Effect of TCM on Number of ICU Admissions, Number of Cases Requiring Any Supplemental Oxygenation and Number of Deaths Among COVID-19 Patients

**Figure 5:**
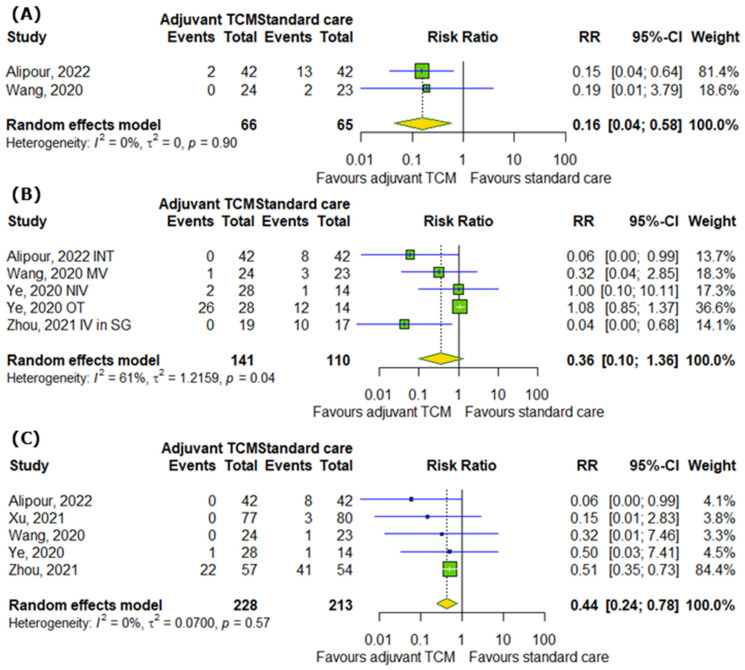
In comparison to standard COVID-19 treatment, the addition of traditional Chinese medicine to standard COVID-19 treatment improves (**A**) the number of ICU admissions, does not affect (**B**) the number of cases requiring any supplemental oxygenation, and decreases (**C**) the number of deaths among COVID-19 patients [14,17,19,27,28]. INT—intubation; MV—mechanical ventilation; NIV—non-invasive ventilation; OT—oxygen therapy; IV in SG—an invasive ventilator in the severe group.

When it comes to the influence of TCM on the number of ICU admissions, the number of cases requiring any supplemental oxygenation, and the number of deaths among COVID-19 patients, our analysis demonstrated that TCM can reduce the risk of ICU admission by 84% (RR = 0.16; 95% CI [0.04; 0.58]; *p* = 0.0053; I^2^ = 0%) and the risk of death by 56% (RR = 0.44; 95% CI [0.24; 0.78]; *p* = 0.0052; I^2^ = 0%), but non-significantly reduces the risk of requiring any supplemental oxygenation (RR = 0.36; 95% CI [0.1; 1.36]; *p* = 0.1318; I^2^ = 61%) (Figure 5A–C).

### 2.6. Effect of TCM on Conversion Rate of SARS-CoV-2 Tests on Particular Days and Time to Viral Assay Conversion

**Figure 6:**
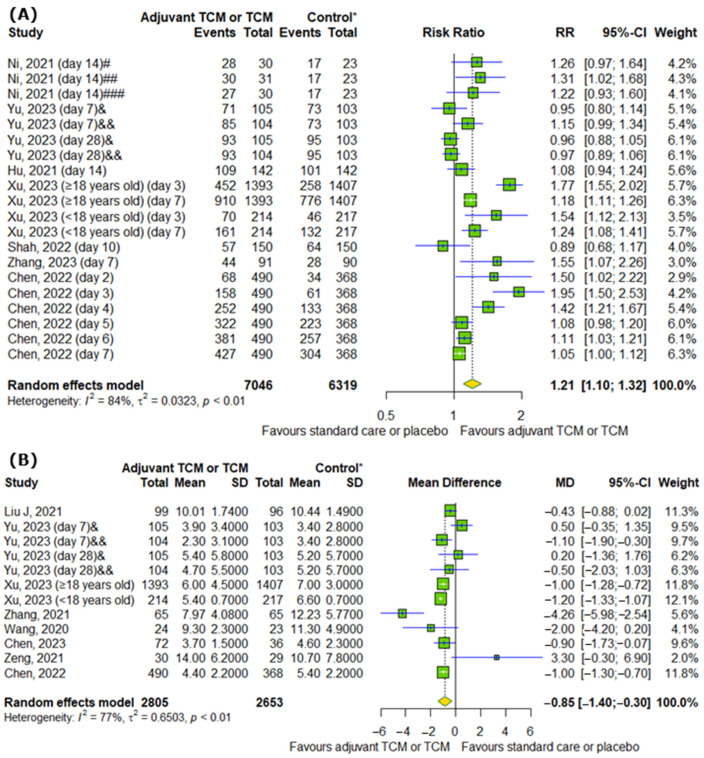
In comparison to standard COVID-19 treatment or placebo, the addition of traditional Chinese medicine to standard COVID-19 treatment or only TCM affects SARS-CoV-2 tests by increasing (**A**) the conversion rate of SARS-CoV-2 tests on particular days and by reducing (**B**) the time to viral assay conversion [8,10,11,13,15,16,17,20,21,23,25,26]. * standard care or placebo; **#** low-dose group; **##** middle-dose group; **###** high-dose group; & Huashi Baidu; && Huashi Baidu and Paxlovid.

Interestingly, TCM positively affects the conversion rate of SARS-CoV-2 tests on particular days (RR = 1.21; 95% CI [1.10; 1.32]; *p* < 0.0001; I^2^ = 84%) (Figure 6A). Moreover, we detected differences between analyzed groups in time to viral assay conversion (MD = −0.85; 95% CI [−1.4; −0.3]; *p* = 0.0025; I^2^ = 77%) (Figure 6B).

### 2.7. Publication Bias

Figure S2 shows the funnel plots for all investigated outcomes, such as time to recovery from fever, severity of dyspnea or breathlessness according to different scales, time to recovery for coughing, including dry and wet coughing, time to recovery for fatigue, changes in respiratory rate, length of hospitalization, hospital discharging rate, number of ICU admissions, number of cases requiring any supplemental oxygenation, number of deaths among COVID-19 patients, conversion rate of SARS-CoV-2 test on particular days, and time to viral assay conversion. Additionally, we performed Peters’ regression test and Egger’s regression test to calculate publication biases for these outcomes. The results showed that there was no evidence of publication bias for the association between TCM and time to recovery from fever (*p* = 0.5081), time to recovery for coughing (*p* = 0.0874), length of hospitalization (*p* = 0.8311), hospital discharging rate (*p* = 0.5634), number of cases requiring any supplemental oxygenation (*p* = 0.7809), number of deaths (*p* = 0.8462), conversion rate of SARS-CoV-2 tests on particular days (*p* = 0.3513), and time to viral assay conversion (*p* = 0.2534). However, publication bias can occur between TCM and severity of dyspnea or breathlessness according to different scales (*p* = 0.0343) and time to recovery for fatigue (*p* = 0.0340). Tests for other outcomes could not be calculated because too few studies were included.

## 3. Discussion

We have carried out a meta-analysis of data collected from 22 randomized clinical trials and demonstrated a positive effect of TCM on COVID-19 symptoms, namely time to recovery from fever, severity of dyspnea or breathlessness according to different scales, time to recovery for coughing, including dry and wet coughing, time to recovery for fatigue, and changes in respiratory rate. Moreover, TCM influences hospitalization duration, ICU admission, mortality, and time to viral assay conversion among COVID-19 patients. Interestingly, TCM positively affected the conversion rate of SARS-CoV-2 tests on particular days. However, TCM did not significantly affect the discharge rates of hospitalized patients on particular days or the requirement of any type of supplemental oxygenation among participants. Of note, our data are consistent with data provided by researchers carrying out similar studies.

In terms of the general efficiency of TCM as a supplementary treatment alongside conventional drugs, most of the studies are univocal. A number of studies report a positive effect of TCM. A network meta-analysis showed that the Maxing Shigan decoction in combination with conventional treatment was more successful in COVID-19 treatment than the conventional treatment [29]. Another study showed that a combination of Chinese herbal medicine and conventional anti-COVID-19 treatment resulted in improvements in multiple parameters related to lungs, fever, COVID-19 severity, and antiviral immune response, in comparison to conventional treatment alone [30]. Similar results were obtained by a number of authors as the addition of varying forms of herbal TCM treatment alleviated COVID-19 symptoms including cough, fever, and fatigue, as well as hampered progression of the disease [31,32,33,34]. On the other hand, some authors reported that subsequent implementation of TCM and conventional treatment improves therapy efficiency and chest CT results, and decreases disease severity, but lacks impacts on COVID-19-associated fever [35]. Interestingly, some authors report that despite some evidence of benefits of TCM in COVID-19 treatment accompanied by relative safety of this approach is apparent. However, the analyzed studies may be affected by a high risk of bias, and include too-small numbers of participants in order to be clinically relevant, and more reliable studies are required to draw a conclusion [36,37].

More evidence of the potentially beneficial effects of TCM in COVID-19 patients can be found in observational studies. In a multicenter, prospective observational study, Hua et al. [38] showed that a kumquat decoction containing kumquat, jiegeng (Platycodonis radix) and two other Chinese herbs was correlated with faster cough relief in comparison to the control group. In a group with moderate COVID-19, without cough and dyspnea symptoms, administration of a Chinese herbal decoction—a mixture of 11 herbs—resulted in quicker viral clearance, measured by negative PCR test results [39]. Furthermore, the efficiency of some TCM approaches has been shown in a number of studies conducted in severely and critically ill COVID-19 patients based on systematic reviews of RCTs and observational studies [40]. Administration of Shenhuang Granule, which comprises *Panax ginseng* C. A. Mey, *Rheum palmatum* L. stem, *Sargentodoxa cuneata* stem, *Taraxacum mongolicum*, *Aconiti Lateralis Radix Praeparata*, and *Whitmania pigra*, in addition to the standard therapy, decreased mortality, the time patients spent in the ICU, and the need for mechanical ventilation, as observed in an observational study in 118 people [41]. In a retrospective cohort study, reduced COVID-19 mortality was also observed by another group of researchers who treated severely and critically ill COVID-19 patients with a TCM decoction, where 94 people were selected for both groups [42]. To date, obtained results may suggest that the administration of TCM might be beneficial, regardless of COVID-19 severity, although due to the high probability of bias in randomization processes and small test populations, the results may not be reliable, and further research on the topic is required [40].

Unfortunately, our meta-analysis has some limitations. Firstly, the studies analyzed used different types of TCM, but due to the small number of included studies, they were analyzed together, as it was not possible to perform subgroup analyses. This may have affected the interpretation of the results and the high heterogeneity of the analyses. Secondly, only one study was conducted outside China, which may have affected the bias of the results. Thirdly, the study included participants with varying degrees of COVID-19 severity, which may also affect the heterogeneity of the analyzed data. In addition, the herbs used in TCM could potentially interact with standard COVID-19 therapy, which could affect treatment. All of this could have affected the interpretation of the results. Taking into consideration the abovementioned results of our study, data presented by other authors of meta-analyses, and finally observational studies, the efficiency of TCM looks promising in the treatment of COVID-19. Moreover, by integrating TCM with standard medicine, the treatment period can be shortened, thereby leading to a reduction in healthcare costs for patients. Despite that, we believe, similarly to many other authors, that further research on the topic is required. The conclusion may be supported by recommendations of the WHO that originated from the “*WHO Expert Meeting on Evaluation of TCM in the Treatment of COVID-19*” [43], where the experts considered up-to-date evidence on the efficiency of TCM in COVID-19 management. The experts highlighted the need for further research, including improving the analysis of data already collected and, most importantly, the need for new, reliable clinical trials. The experts stated that well-planned studies that include properly assessed key factors such as sample size, randomization, and blinding are required. Notably, the trials should be based on the worldwide cooperation of WHO Member States, including hospitals outside of China.

## 4. Materials and Methods

### 4.1. Search Strategy

This meta-analysis was conducted in accordance with the PRISMA (Preferred Reporting Items for Systematic Reviews and Meta-Analyses) guidelines [44]. Databases such as PubMed, Embase, and the Cochrane Central Register of Controlled Trials were searched. Literature published until 5 April 2024 was included. The following keywords were used: “Traditional Chinese medicine”, “acupuncture”, “moxibustion”, “cupping”, “tai chi”, “Chinese herbal medicine”, “COVID-19”, “coronavirus infection”, and “SARS-CoV-2”. The detailed search steps are shown in Table S2.

### 4.2. Selection of Studies and Data Extraction

Inclusion criteria included only articles written in English of RCTs examining the efficacy of TCM only or in addition to COVID-19 treatment compared to placebo or standard care in the treatment of COVID-19. Articles should have been published before 5 April 2024 and included the following endpoints: time to recovery from fever, severity of dyspnea or breathlessness according to different scales, time to recovery for coughing, including dry and wet coughing, time to recovery for fatigue, changes in respiratory rate, length of hospitalization, hospital discharging rate, number of ICU admissions, number of cases requiring any supplemental oxygenation, number of deaths among COVID-19 patients, conversion rate of SARS-CoV-2 tests on particular days, and time to viral assay conversion. Exclusion criteria were as follows: articles not written in English and not containing the endpoints.

Continuous data were converted into means (SD):

- If the data were presented as a median with quartiles [median (Q1, Q3)] or a median (range), the value was converted according to the method presented by Luo et al. [45] and Wan et al. [46] using available calculators without checking the skewness.
- If the data were presented as a mean (95% confidence intervals), the value was converted according to the *Cochrane Handbook for Systematic Reviews of Interventions* [47] using the formula $SD=\sqrt{N}\times (upper\;limit-lower\;limit)/3.92.$

If the number of cases was a percentage, it was converted to whole numbers in accordance with rounding rules.

### 4.3. Quality Evaluation

The quality of trials was assessed using the Cochrane Collaboration’s tool for assessing the risk of bias in randomized trials [48]. The following criteria were used: random sequence generation, allocation concealment, blinding of participants and personnel, blinding of outcome assessment, incomplete outcome data, selective reporting, and other bias (assessing at 3 levels such as low, high, or unclear risk).

### 4.4. Statistical Analysis

Statistical analysis of data was performed in R (version 4.2.2). To compare the effects of TCM on COVID-19 in the experimental group compared with the control group, the relative risk (RR) with 95% confidence intervals (CIs) was calculated for dichotomous outcomes, while mean differences or standardized mean differences with 95% CIs were calculated for continuous outcomes. A random effects model was utilized to calculate effect sizes. I^2^ statistics were employed to assess the heterogeneity among the studies: I^2^ < 40% may not be important; 30% < I^2^ < 60% means moderate heterogeneity; 50% < I^2^ < 90% means substantial heterogeneity; I^2^ > 75% means considerable heterogeneity [49]. To assess publication bias, funnel plots, Peters’ regression test (for dichotomous outcomes), and Egger’s regression test (for continuous outcomes) were used. The results of this meta-analysis were deemed to be statistically significant at *p* < 0.05.

## 5. Conclusions

In conclusion, the results of our study suggest that traditional Chinese medicine may be beneficial when used in combination with conventional treatment for SARS-CoV-2. However, the lack of high-quality clinical trials, difficulties in standardizing the therapy, and possible drug interactions mean that its effectiveness remains uncertain. It should be noted that further randomized, controlled trials with larger sample sizes and from more countries are needed to provide more robust evidence to support this hypothesis.

## Figures and Tables

**Table 1:** Basic characteristics of included studies.

Studies	Study Design	Participants	Age, Mean (SD)	% of Female	Treatment	The Duration of Treatment	References
Alipour et al., 2022	randomized, three-arm trial, with blinded outcome assessment	18–75-year-old patients with moderate to severe SARS-CoV-2	I: 52.74 (12.72) C: 57.90 (12.15)	I: 59.53% C: 66.67%	I: acupuncture once daily + conventional treatment C: conventional treatment	3–7 days	[28]
Zhao et al., 2021	prospective, single-centered, cluster-randomized, parallel-controlled, unblinded clinical trial	patients newly diagnosed with mild COVID-19	I: 50.6 (16.1) C: 49.4 (15.2)	I: 51.4% C: 49.5%	I: 10 g of Huashi Baidu granule twice daily + conventional treatment C: conventional treatment	7 days	[7]
Liu J et al., 2021	single-center, open-label, randomized controlled trial	patients aged 18 to 75 with COVID-19	I: 55.47 (10.16) C: 55.8 (10.16)	I: 63.6% C: 61.5%	I: 10 g Huashi Baidu granule (Q-14) twice daily + standard care C: standard care	14 days	[8]
Liu ST et al., 2021	single-center, parallel-arm, randomized controlled trial	patients aged 20–80 diagnosed with severe COVID-19	I: 50.0 (4.2) C: 53.6 (5.2)	I: 60.9% C: 53.2%	I: QARP consisting of qigong exercise (Liu Zi Jue) and acupressure therapy twice daily + standard therapies C: standard therapies	until the day of discharge	[9]
Ni et al., 2021	randomized, open-label, parallel-controlled, multicenter trial	18 years or older with COVID-19	I1: 52.67 (15.41) I2: 54.94 (15.94) I3: 52.47 (16.34) C: 51.53 (20.14)	I1: 58.9% I2: 45.9% I3: 54.2% C: 57.6%	I1 (low-dose group): 20 mL of Shuanghuanglian three times daily + standard therapy I2 (middle-dose group): 40 mL of Shuanghuanglian three times daily + standard therapy I3 (high-dose group): 60 mL of Shuanghuanglian three times daily + standard therapy C: standard therapy	14 days	[10]
Yu et al., 2023	prospective, single-center, three-arm, randomized trial	aged 18 years and older, hospitalized with symptomatic COVID-19 infection with Omicron (B.1.1.529) and high risk for progression to severe diseases	I1: 69.64 (16.1) I2: 71.63 (15.32) C: 67.01 (16.34)	I1: 49% I2: 47% C: 51%	I1: 137 g of Huashi Baidu (HSBD) twice daily for 7 days I2: combination of HSBD and Paxlovid C: Paxlovid (300 mg of Nirmatrelvir plus 100 mg of Ritonavir) every 12 h for 5 days	7 or 5 days	[11]
Zhang et al., 2022	randomized, open-label, blank-controlled, multicenter trial	patients ≥18 years of age with mild and common-type COVID-19	I: 49.56 (14.88) C: 52.81 (14.83)	I: 68.06% C: 66.67%	I: Lianhua Qingke administration (4 tablets, thrice daily) + routine treatment C: routine treatment	14 days	[12]
Hu et al., 2021	prospective, open-label, randomized controlled trial	patients aged 18 years or greater with symptomatic COVID-19	I: 50.4 (15.2) C: 51.8 (14.8)	I: 44.4% C: 50%	I: combination of Lianhuaqingwen capsules (4 capsules thrice daily) + usual treatment C: usual treatment	14 days	[13]
Xu et al., 2021	randomized, open-labeled, multicenter, controlled trial	patients ≥18 years of age with symptomatic COVID-19	I: 49.1 (15.7) C: 50.4 (16.0)	I: 44.2% C: 45%	I: Reduning injection once a day + routine treatment C: routine treatment	2 weeks	[14]
Xu et al., 2023 (≥18 years old)	prospective, open-label, randomized controlled trial	patients aged 18–80 years with asymptomatic and mild COVID-19	I: 44.9 (16.33) C: 44.3 (17.81)	I: 41.7% C: 40.4%	I: oral Reyanning (RYN) mixture (20 mL, 4 times a day) + standard care C: standard care	7 days	[15]
Zhang et al., 2021	multicenter, prospective, open-label and randomized controlled trial	patients at least 18 years of age with mild or moderate symptomatic COVID-19	I: 44.31 (13.45) C: 48.25 (14.22)	I: 50.8% C: 56.9%	I: Xiyanping injection was given at a weight-based dose of 10 mg/kg once per day, with a maximum daily dosage not to exceed 500 mg + standard care C: standard symptomatic treatments	7–14 consecutive days	[16]
Wang et al., 2020	two-arm, randomized, controlled phase I/II trial	patients from “suspected COVID-19 patients”	I: 46.8 (14.4) C: 51.4 (17.6)	I: 41.7% C: 47.8%	I: 19.4 g of Keguan-1 (meaning anti-coronavirus 1 in Chinese) twice daily + control therapy C: control therapy	2 weeks	[17]
Li et al., 2022	randomized clinical trial	individuals with asymptomatic COVID-19	I: 53 (13) C: 56 (17)	I: 35% C: 36%	I: orally one bag (1.5 g/bag) of ginger supplements twice daily + general medical care C: general medical care	until discharged from the hospital	[18]
Ye et al., 2020	open-label, pilot, randomized trial	adult patients (≥18 years) with severe COVID-19	I: 62.32 (12.11) C: 57.54 (16.47)	I: 93% C: 71%	I: Chinese herbal medicine according to the NHC NATCM China guidelines; each unit of formula yielded 400 mL of decoction, divided into two equal portions, administered 200 mL orally twice daily + standard care C: standard care	7 days	[19]
Chen et al., 2023	single-center, open-label, parallel-group randomized controlled clinical trial, with an allocation ratio of 2:1	age 3–18 years, with mild COVID-19	I: 8 (4.54) C: 9 (3.09)	I: 40.3% C: 55.6%	I: Huashi Baidu granule (HSBDG) with a dose of 2.5 g for ages 3–6 years, 5 g for ages 7–12 years, and 10 g for ages 13–18 years, twice daily C: compound pholcodine oral solution with a dose of 5 mL for ages 3–6 years and 10 mL for ages 7–18 years, three times daily	5 days	[20]
Shah et al., 2022	phase 2–3, double-blind, randomized, placebo-controlled clinical trial	age range of 18–75 years with mild symptomatic non-hospitalized SARS-CoV-2 infection	I: 38.89 (11.87) C: 38.02 (11.47)	I: 39.33% C: 34.67%	I: Jinhua Qinggan granules at an oral dose of 5 g (1 sachet) three times a day C: placebo	10 days	[21]
Zhang et al., 2023	prospective randomized controlled trial	age ≥ 14 years, with asymptomatic infection and mild Omicron BA.2	I: 41.09 (14.69) C: 40.06 (13.52)	I: 57.14% C: 54.44%	I: Liushen pill, three sublingual pills and seven oral pills at a time, three times a day and Maizao decoction one dose a day divided into two doses C: placebo (Maizao decoction)	7 days; if the patient tested negative before this, the treatment was ended early	[22]
Xia et al., 2022	randomized controlled trial	age 18–80 years with moderate COVID-19	I: 55.1 (13.7) C: 56.3 (11.9)	I: 56.3% C: 60.0%	I: one total dose of TCM daily, taken twice depending on conditions + Western medicine treatment C: Western medicine treatment	NA	[23]
Xu et al., 2023 (<18 years old)	prospective, open-label randomized controlled trial	1–17 years of age with asymptomatic or mild COVID-19	I: 11.65 (5.22) C: 12.18 (5.6)	I: 39.4% C: 41.9%	I: oral Reyanning Mixture (RYN), dosages for children were as follows: 3 years old, 5 mL, 3 times a day; 4–6 years, 10 mL, 3 times a day; 7–14 years, 15 mL, 3 times a day; 15–17 years, 20 mL, 4 times a day + standard care C: standard care	7 days	[24]
Zeng et al., 2021	open-label randomized controlled trial	age of 18–85 years, symptomatic, mild, and moderate COVID-19	I: 50.7 (12.3) C: 53.3 (15.8)	I: 36.7% C: 27.6%	I: 200 mL of Maxingshigan-Weijing decoction, orally 2 times daily + routine supportive care C: routine supportive care	14 days	[25]
Chen et al., 2022	prospective, double-blind, randomized, placebo-controlled trial	patients 18 to 80 years old with mild symptoms of COVID-19	I: 46.9 (17.84) C: 46.25 (17.12)	I: 38.37% C: 51.09%	I: JingYinGuBiao (JYGB) granules orally for 15 g twice daily C: placebo orally for 15 g twice daily	7 days	[26]
Zhou et al., 2021	randomized, controlled, multicenter, open-label trial	patients ≥18 years of age with severe/critical COVID-19	NA	NA	I: Shenhuang Granule (SHG) was dissolved in warm water and taken orally, with a dosing regimen of two sachets per day (after 14 days, SHG was given twice daily until death or discharge) + standard care C: standard care	14 days and more until death or discharge	[27]

NA—not applicable; I—intervention group; C—control group; the NHC NATCM—the National Health Commission and the National Administration of Traditional Chinese Medicine; SD—standard deviation.

## Data Availability

The original contributions presented in this study are included in the article/Supplementary Materials.

## Supplementary Materials

The following supporting information can be downloaded at https://www.mdpi.com/article/10.3390/ph18030357/s1, Table S1: Detailed description and TCM formulas used in the included studies. Figure S1: Risk of bias of included studies. Figure S2: Funnel plots for the associations between traditional Chinese medicine and COVID-19. Table S2: Search strategy in PubMed, Embase, and the Cochrane Central Register of Controlled Trials.

## Abbreviations

The following abbreviations are used in this manuscript:

TCM	Traditional Chinese medicine
ICU	Intensive care unit
CI	Confidence interval
RR	Relative risk
SD	Standard deviation
RCTs	Randomized controlled trials
