# Supplementary material for: Effect of Traditional Chinese Medicine on COVID-19 Treatment: A Meta-Analysis of Randomized Clinical Trials
Source: Pharmaceuticals (Basel). 2025 Mar 2;18(3):357. doi: 10.3390/ph18030357 (PMC11945379; doi:10.3390/ph18030357)
Supplement: Supplementary file 1 [file pharmaceuticals-18-00357-s001.zip › pharmaceuticals-3492064-supplementary.pdf]

Table S1. Detailed description and TCM formulas used in the included studies.

Studies	TCM description	References
Alipour et al., 2022	Standard needling method (without electrical stimulation and by using disposable tube needles) was performed with 0.25 mm × 40 mm, single-use, sterile, stainless-steel needles. Selected acupoints included: Governor Vessel-20 (GV20; Baihui), Lung-5 (LU5; Chize), Lung-7 (LU7; Lieque), Large Intestine-4 (LI4; Hegu), Liver-3 (LR3; Taichong), Liver-14 (LR14; Qimen), Conception Vessel-12 (CV12; Zhongwan), Conception Vessel-17 (CV17; Tanzhong) and Stomach-36 (ST36; Zusanli).	[28]
Zhao et al., 2021	The daily dosage of the Huashi Baidu granule was converted from CM decoction pieces: *Ma-huang* (Herba Ephedrae) 6 g, *Ku-xing-ren* (Armeniacae Semen Amarum) 9 g, *Shi-gao* (Gypsum Fibrosum) 15 g, *Gan-cao* (Glycyrrhizae Radix et Rhizoma) 3 g, *Huo-xiang* (Pogostemonis Herba) 10 g, *Hou-pu* (Magnoliae Officinalis Cortex) 10 g, *Cang-zhu* (Atractylodis Rhizoma) 15 g, Cao-guo (Tsaoko Fructus) 10 g, *Fa-ban-xia* (Pinelliae Rhizoma Praeparatum) 9 g, *Fu-ling* (Poria) 15 g, *Sheng-da-huang* (Rhei Radix et Rhizoma) 5 g, *Sheng-huang-qi* (Astragali Radix) 10 g, *Ting-li-zi* (Descurainiae Semen Lepidii Semen) 10 g, *Chi-shao* (Paeoniae Radix Rubra) 10 g.	[7]
Liu J et al., 2021	Q-14, also known as Huashi Baidu granule, is a compound granule composed of 14 Chinese herbs, including *Mahuang* (Herba Ephedrae), *Kuxingren* (Armeniacae Semen Amarum), *Shigao* (Gypsum Fibrosum), *Gancao* (Glycyrrhizae Radix et Rhizoma), *Huoxiang* (PogostemonisHerba), *Houpu* (Magnoliae Officinalis Cortex), *Cangzhu* (Atractylodis Rhizoma), *Caoguo* (TsaokoFructus), *Fabanxia* (Pinelliae Rhizoma Praeparatum), *Fuling* (Poria), *Shengdahuang* (Rhei Radix et Rhizoma), *Shenghuangqi* (Astragali radix), *Tinglizi* (Descurainiae Semen Lepidii Semen), *Chishao* (Paeoniae Radix Rubra).	[8]
Liu ST et al., 2021	QARP (qigong exercise and acupressure rehabilitation program) was performed twice daily at 10 am and 4 pm. The participants were required to pronounce "Xu, He, Hu, Si, Chui, Xi" in a relaxed condition for 12 cycles. Each treatment lasted 20 min for a total of 40 min every day.<br>The acupressure treatment was performed by a physical therapist every day after reaching a consensus on the participants' acupuncture points, pressure levels, and duration. The acupoints were Feishu (BL13), Danzhong (RN17), and Zhongfu (LU1), which are related to the lung viscera.	[9]
Ni et al., 2021	Shuanghuanglian oral liquid, a traditional Chinese patent medicine containing extracts of three herbs, including *Lonicera japonica* Thunb., *Scutellaria baicalensis* Georgi, and *Forsythia suspense* (Thunb.).	[10]

Yu et al., 2023	The composition of Huashi Baidu granule includes 14 Chinese herbs, such as *Mahuang* (Herba Ephedrae), *Xingren* (Armeniacae Semen Amarum), *Shigao* (Gypsum Fibrosum), *Gancao* (Glycyrrhizae Radix et Rhizoma), *Huoxiang* (Pogostemonis Herba), *Houpu* (Magnoliae Officinalis Cortex), *Cangzhu* (Atractylodis Rhizoma), *Caoguo* (Tsaoko Fructus), *Fabanxia* (Pinelliae Rhizoma Praeparatum), *Fuling* (Poria), *Shengdahuang* (Rhei Radix et Rhizoma), *Shenghuangqi* (Astragali radix), *Tinglizi* (Descurainiae Semen Lepidii Semen), *Chishao* (Paeoniae Radix Rubra).	[11]
Zhang et al., 2022	Lianhua Qingke tablets is mainly composed of thirteen herbs, including *Mahuang* (stem, Herba Ephedra), *Sangbaipi* (root bark, Morus Alba L.), *Shigao* (Gypsum fibrosum), *Huangqin* (root, Scutellaria baicalensis), *Kuxingren* (semen, armeniacae amarum), *Lianqiao* (fruit, Forsythia suspensa), *Banxia* (stem, Pinellia ternate), *Zhebeimu* (bulb, Fritillaria thunbergii), *Qianhu* (root, Peucedanum praeruptorum), *Niubangzi* (fruit, Fructus arctii), *Jinyinhua* (flos, Lonicera japonica Thunb.), *Dahuang* (root, Rhei Radix et Rhizoma), and *Chenpi* (pericarpium, Citrus reticulata Blanco.).	[12]
Hu et al., 2021	The major ingredients of Lianhuaqingwen capsule consisted of *Forsythia suspensa, Lonicera japonica, Ephedra sinica, Isatis indigotica, Pogostemon cablin, Rheum palmatum, Glycyrrhiza uralensis, Dryopteris crassirhizoma, Rhodiola crenulata, Houttuynia cordata, Prunus sibirica,* gypsum and 1-menthol.	[13]
Xu et al., 2021	Reduning injection is composed of three TCM herbs: *Artemisia annua, Lonicera japonica Thunb,* and *Gardenia jasminoides Ellis.* 20 mL of Reduning injection was diluted with 250 mL of saline and then intravenously infused.	[14]
Xu et al., 2023 (≥18 years old)	Reyanning mixture was composed of *Taraxacum, Polygonum cuspidatum, Patrinia villosa,* and *Scutellaria barbata.*	[15]
Zhang et al., 2021	Xiyanping (XYP) injection, is a proprietary Chinese medicine prepared from herbaceous plant *A. paniculate*. The bioactive ingredient, andrographolide, was extracted from the aerial part of A. paniculate using ethanol, and was then sulfonated through sulfonation reaction to generate XYP preparation. The concentration of andrographolide sulfonation product in XYP injection is 25 mg/ml.	[16]
Wang et al., 2020	The new formula was derived from 3 different formulae, Yinqiao Powder (银翘散), Sangju Drink (桑菊饮), and Sanren Decoction (三仁汤), named "Keguan-1" (meaning anti-coronavirus 1 in Chinese) with 7 components: *Lonicera japonica* Thunb. (Jinyinhua, lot. 19040301) 30 g, *Forsythia suspensa* (Thunb.) Vahl, (Lianqiao, lot. 19040221) 30 g, *Morus alba L.* (Sangye, lot. 19045321) 15 g, *Chrysanthemum morifolium* Ramat. (Juhua, lot. 19040811) 10 g, *Coix lacryma-jobi* L. *var. mayuen* (Roman.) Stapf, *Yiyiren*, lot. 19025161) 30 g, *Fritillaria thunbergii* Miq. (Zhebeimu,	[17]

	lot. 19041161) 15 g, and *Prunus armeniaca* L. var. *ansu* Maxim. (Kuxingren, lot. 19045591) 9 g.	
Li et al., 2022	The ginger supplements were provided in the form of ginger powder packed individually (1.5 g/bag), participants in the ginger supplement group were asked to mix it with hot water and take it orally.	[18]
Ye et al., 2020	Based on symptom-based syndrome differentiation using traditional Chinese medicine principles, the included patients in the GC plus group were divided into the following two syndromes: Lung Blocked by Epidemic Toxin and Inner Blocking Causing Collapse.<br>For patients presented with Lung Blocked by Epidemic Toxin syndrome, the modified maxingshigan formula were used; the formula is composed of the following herbs: *xingren* (stir-baked Semen Armeniacae Amarum), 10 g; *shengshigao* (raw Gypsum Fibrosum), 30 g; *gualou* (Trichosanthis Fructus) 30g; *shengdahuang* (Rhei Radix et Rhizoma) 6g (added at the end of decoction preparation); *shengmahuang* (raw Herba Ephedrae), 6g; *zhimahuang* (honey-fried Herba Ephedrae), 6 g; *tinglizi* (Descurainiae Semen) 10g, *taoren* (Persicae Semen) 10g; *caoguo* (Tsaoko Fructus) 6g, *binlang* (Arecae Semen) 10g; *cangzhu* (Atractylodis Rhizoma) 10g.<br>For patients presented with Inner Blocking Causing Collapse syndrome, the modified shenfutang formula will be used together with Proprietary Chinese Medicine pomanders. Suhexianwan (3g, twice a day with herbal decoction) or Angongniuhuangwan (one pomander, twice a day with herbal decocation). Modified Shengfutang formula is composed of the following herbs: *rensheng* (Ginseng Radix et Rhizoma), 15g; *Heishunpian* (Aconiti Lateralis Radix Praeparata), 10g (cook prior to mixture with other herbs); *shanzhuyu* (Evodiae Fructus), 15g.	[19]
Chen et al., 2023	Huashi baidu granule were composed of *Houpu* (Magnoliae Officinalis Cortex), *Cangzhu* (Atractylodis Rhizoma), *Caoguo* (TsaokoFructus), *Mahuang* (Herba Ephedrae), *Kuxingren* (Armeniacae Semen Amarum), *Shigao* (Gypsum Fibrosum), *Gancao* (Glycyrrhizae Radix et Rhizoma), *Huoxiang* (PogostemonisHerba), *Fabanxia* (Pinelliae Rhizoma Praeparatum), *Fuling* (Poria), *Shengdahuang* (Rhei Radix et Rhizoma), *Shenghuangqi* (Astragali radix), *Tinglizi* (Descurainiae Semen Lepidii Semen), and *Chishao* (Paeoniae Radix Rubra).	[20]
Shah et al., 2022	Jinhua Qinggan granules were synthesized from the two TCM formulae, namely, Ma Xing Shi Gan Decoction and Yin Qiao San Decoction, containing 11 herbs including *Honeysuckle, Ephedrae Herba, Armeniacae Semen Amarum, Scutellariae Radix, Forsythiae Fructus, Fritillariae thunbergii Bulbus, Anemarrhenae Rhizoma, Arctii Fructus, Artemisiae annuae Herba, Menthae haplocalycis Herba, Glycyrrhizae Radix et Rhizoma* along with a traditional Chinese mineral medicine, and Gypsum Fibrosum.	[21]

Zhang et al., 2023	Liushen pill is composed of musk, bezoar, toad, realgar, and six other medicinal herbs; however, the exact formulation is a national top-secret formula.<br>Maizao decoction is composed of 15 g fried malt, 10 g jujube, 200 ml decoction.	[22]
Xia et al., 2022	The TCM treatment included combinations for the following conditions: (a) Cold-damp constraint in the lung pattern , recommended prescription: Mahuang (*Herba Ephedra Sinica*) 6 g, Kuxingren (*Semen Armeniacae Amarum*) 15 g, Shigao (*Gypsum Fibrosum*) 30 g, Yiyiren (*Semen Coicis*) 30 g, Cangzhu (*Rhizoma Atractylodis Lanceae*) 10 g, Huoxiang (*Herba Agastaches Rugosa*) 15 g, Qinghao (*Herba Artemisiae Annuae*) 12 g, Huzhanggen (*Radix Polygoni Cuspidati*) 20 g, Mabiancao (*Herba Verbenae Officinalis*) 30 g, Lugen (*Rhizoma Phragmitis*) 30 g, Zhizi (*Fructus Gardeniae*) 15 g, Gualou (*Fructus et Semen Trichosanthis*) 30g, Dahuang (*Radix Et Rhizoma Rhei Palmati*) 6g (decocted later), Juhong (*Exocarpium Citri Rubrum*) 15 g, and Gancao (*Radix Glycyrrhizae*) 10 g; (b) Damp-heat accumulation in the lung pattern, recommended prescription: Binglang (*Semen Arecae*) 10 g, Caoguo (*Fructus Tsaoko*) 10 g, Houpu (*Cortex Magnoliae Officinalis*) 10 g, Zhimu (*Rhizoma Anemarrhenae*) 10 g, *Huangqin (Radix Scutellariae Baicalensis)* 10 g, Chaihu (*Radix Bupleuri Chinensis*) 10 g, Chishao (*Radix Paeoniae Rubra*) 10 g, Lianqiao (*Fructus Forsythiae Suspensae*) 15 g, Qinghao (*Herba Artemisiae Annuae*) 10 g (decocted later), Cangzhu (*Rhizoma Atractylodis Lanceae*) 10 g, and Gancao (*Radix Glycyrrhizae*) 5 g; (c) Cold-damp obstructing the lung pattern, recommended prescription: Cangzhu (*Rhizoma Atractylodis Lanceae*) 15 g, Chenpi (*Pericarpium Citri Reticulatae*) 10 g, Houpu (*Cortex Magnoliae Officinalis*) 10 g, Huoxiang (*Herba Agastaches Rugosa*) 10 g, Caoguo (*Fructus Tsaoko*) 6 g, Mahuang (*Herba Ephedra Sinica*) 6 g, Qianghuo (*Rhizoma et Radix Notopterygii*) 10 g, Shengjiang (*Rhizoma Zingiberis Recens*) 10 g, and Binglang (*Semen Arecae*) 10 g. The recommended dosing was: one total dose daily, boiled in 400 mL water and taken twice in the morning and evening.	[23]
Xu et al., 2023 (<18 years old)	Each 1,000 mL of Reyanning Mixture is made from 372 g *Taraxacum mongolicum* Hand.-Mazz., 372 g *Polygonum cuspidatum* Siebold & Zucc., 372 g *Sonchus arvensis* L., and 186 g *Scutellaria barbata* D. Don.	[24]
Zeng et al., 2021	Maxingshigan-Weijing decoction (MWD) consists of 10 g of Herba Ephedra (Mahuang), 10 g of Amygdalus Communis Vas (Xingren), 45 g of Gypsum Fibrosum (Shigao), 30 g of Rhizoma phragmitis (Lugen), 20 g of peach kernel (Taoren), 20 g of winter melon kernel (Dongguaren), 30 g of Trichosanthes Kirilowii Maxim (Gualou), 12 g of Pericarpium Citri Reticulatae (Chenpi), 12 g of Rhizoma Pinelliae (Jiangbanxia), 12 g of caulis bambusae in taeniis (Zhuru), 30 g of	[25]

	semen lepidii (Tingliz), 15 g of semen lepidii (Shichangpu), 10 g of curcuma zedoary (ezhu) and 5 g of Radix Glycyrrhizae (Gancao).	
Chen et al., 2022	JingYinGuBiao formula was composed of 10 herbs: 9g jinyinhua (Lonicera japonica Thunb), 9g jingjie (Herba Schizonepetae), 12g huangqi (Astragalus propinquus Schischkin), 9g fangfeng (Saposhnikovia divaricate), 9g huoxiang (Agastache rugosus), 9g banlangen (Isatis Root), 6g jiegeng (Platycodon Grandiflorum), 15g lugen (rhizoma phragmitis), 9g baishu (Atractylodes macrocephala Koidz), and 9g gancao (GlycyrrhizauralensisFisch).<br>The TCM placebo was used as control in this study, which contained 1g huoxiang and 1g gancao in order to have a similar color and taste (brown and bitter) with JYGB.	[26]
Zhou et al., 2021	The Shenhuang Granule is a formulation of the following raw herbs: 50 g of *Panax ginseng C. A. Mey* (*Renshen*) root, 40 g of *Rheum palmatum L.* stem (*Dahuang*), 30g of *Sargentodoxa cuneata* stem (*Hongteng*), 30 g of *Taraxacum mongolicum* whole plant (*Pugongying*), 50 g of *Aconiti Lateralis Radix Praeparata* stem (*Fuzi*) and 6g of Whitmania pigra Whitman (*Shuizhi*) whole organism. After a series of extraction and manufacturing processes, the final product is a concentrated granule which is 1:5 of the raw herbs.	[27]

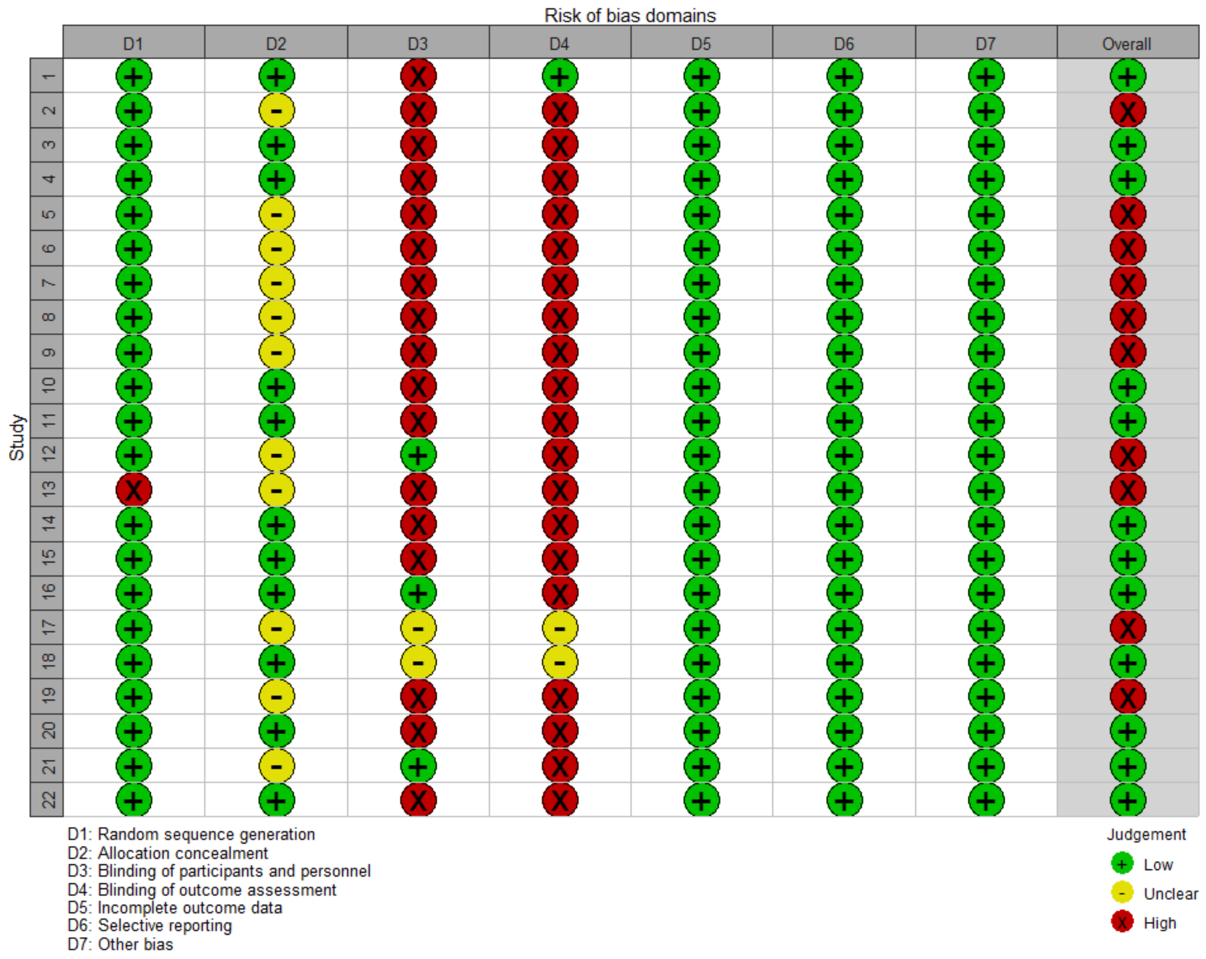

Figure S1. Risk of bias of included studies.

1: Alipour et al., 2022 [28]; 2: Zhao et al., 2021 [7]; 3: Liu et al., 2021 [8]; 4: Liu et al., 2021 [9]; 5: Ni et al., 2021 [10]; 6: Yu et al., 2023 [11]; 7: Zhang et al., 2022 [12]; 8: Hu et al., 2021 [13]; 9: Xu et al., 2021[14]; 10: Xu et al., 2023 [15]; 11: Zhang et al., 2021 [16]; 12: Wang et al., 2020 [17]; 13: Li et al., 2022 [18]; 14: Ye et al., 2020 [19]; 15: Chen et al., 2023 [20]; 16: Shah et al., 2022 [21]; 17: Zhang et al., 2023 [22]; 18: Xia et al., 2022 [23]; 19: Xu et al., 2023 [24]; 20: Zeng et al., 2021 [25]; 21: Chen et al., 2022 [26]; 22: Zhou et al., 2021 [27].

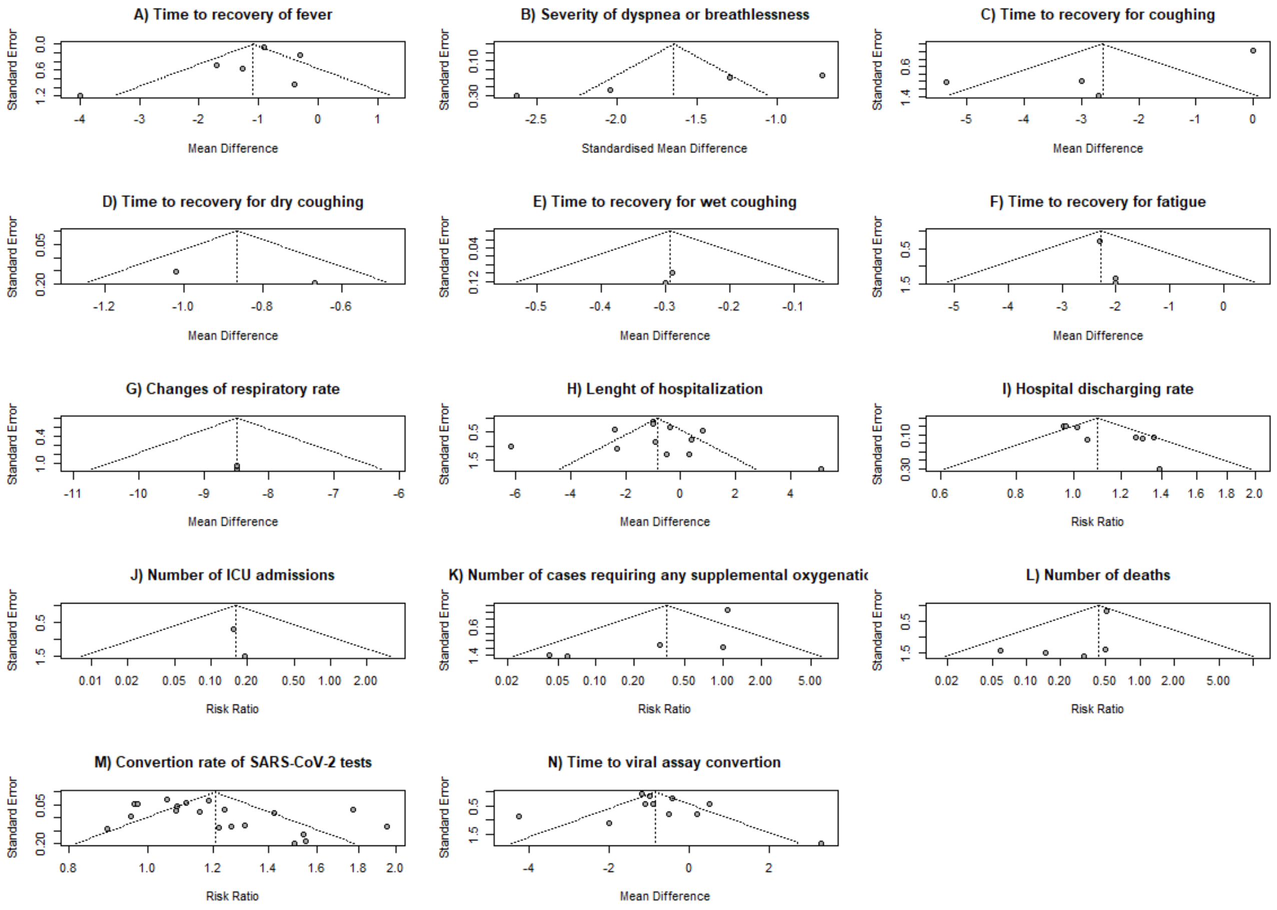

Figure S2. Funnel plots for the associations of between traditional Chinese medicine and COVID-19.

Table S2. Search strategy in PubMed, Embase and the Cochrane Central Register of Controlled Trials.

**Pubmed**		
Set	Searches	Results
#1	((Traditional Chinese medicine) OR (acupuncture) OR (moxibustion) OR (cupping) OR (tai chi) OR (Chinese herbal medicine)) AND ((COVID-19) OR (coronavirus infection) OR (SARS-CoV-2))	3300
**EMBASE**		
#1	'acupuncture'	68067
#2	'traditional Chinese medicine'	150902
#3	'moxibustion'	16409
#4	'cupping'	3433
#5	'tai chi'	4872
#6	'Chinese herbal medicine'	10193
#7	'COVID-19'	416959
#8	'coronavirus infection'	17348
#9	'SARS-CoV-2'	176570
#10	#1 OR #2 OR #3 OR #4 OR #5 OR #6	218655
#11	#7 OR #8 OR #9	458169
#12	#10 AND #11	2943
**The Cochrane Central Register of Controlled Trials**		
#1	Traditional Chinese medicine	24883
#2	acupuncture	22054
#3	moxibustion	6668
#4	cupping	982
#5	tai chi	2171
#6	Chinese herbal medicine	5847
#7	COVID-19	19811
#8	coronavirus infection	3958
#9	SARS-CoV-2	564
#10	#1 OR #2 OR #3 OR #4 OR #5 OR #6	47773
#11	#7 OR #8 OR #9	20114
#12	#10 AND #11	356
